# Androgen Stimulation of PCA3 and miR-141 and Their Release from Prostate Cancer Cells 

**DOI:** 10.22074/cellj.2015.494

**Published:** 2015-01-13

**Authors:** Ugur Gezer, Duygu Tiryakioglu, Elif Bilgin, Nejat Dalay, Stefan Holdenrieder

**Affiliations:** 1 Department of Basic Oncology, Institute of Oncology, Istanbul University, Istanbul, Turkey; 2 Insitute of Clinical Chemistry and Pharmacology, University of Bonn, Bonn, Germany

**Keywords:** Androgens, Culture Media, Mirn141 microRNA, Prostate Cancer, Prostate Cancer Antigen 3

## Abstract

**Objective:**

Prostate cancer antigen 3 (PCA3) and microRNA-141 (miR-141) are emerging molecules in prostate cancer (PCa) pathogenesis and have been shown to be involved in androgen signaling. In this original research, we designed an experimental cell model with androgen-sensitive LNCaP cells to comparatively assess the extent of androgen responsiveness of PCA3-mRNA and miR-141 along with prostate specific antigen (PSA)mRNA and their release into culture medium. These molecules were also measured in the plasma of the patients with early PCa which is considered to be analogous to androgenresponsive cells.

**Materials and Methods:**

In this experimental study, LNCaP cells were exposed to androgen ablation for 48 hours and treated then with dihydrotestosterone (DHT) for 24 hours. Expression of all three RNA molecules in cells, culture medium or plasma was measured by quantitative polymerase chain reaction (qPCR).

**Results:**

Our results show that DHT differentially affects the expression of these molecules. PCA3 was the most evidently induced molecule (up to 400-fold, p<0.001), while the effect was moderate for PSA-mRNA (up to 30-fold, p<0.001). In contrast, the stimulation of miR-141 was much weaker (up to 1.5-fold, p>0.05). With regard to the release into culture medium, a similar picture was observed except for PCA3. PCA3 was below the detection level despite its high stimulation. DHT treatment led to a significant release of PSA-mRNA (up to 12-fold). Similar to its induction pattern in LNCaP cells, miR-141 was released at a limited quantity into the medium (up to 1.7- fold, p=0.07). In plasma, only PCA3 differed significantly between the patients and healthy subjects (p=0.001).

**Conclusion:**

Our findings indicate that PCa-related RNA molecules respond differentially to androgen stimulation suggesting differential regulation by androgens.

## Introduction

Prostate cancer (PCa) is one of the most prevalent malignant diseases among men in Western countries (1). Early stage prostate cancer depends on androgens for growth (2). The most effective systemic treatment for this hormonesensitive cancer has been androgen deprivation therapy (ADT). In some patients the tumor evolves to castration resistant PCa (CRPC) which is defined as apparent tumor growth in the presence of castrating levels of androgens (˂50 ng/ml). The serum prostate-specific antigen (PSA) test has been used for the detection of PCa at early stages and is also routinely utilized to monitor PCa recurrence after therapy. However, this has limited accuracy in predicting treatment outcomes and making clinical decisions (3) necessitating urgently the need for additional prognostic markers for PCa.

Prostate cancer antigen 3 (PCA3), a long noncoding RNA (lncRNA), is an emerging molecule in PCa. It is highly expressed in PCa cell lines and primary tumor specimens (4, 5). Urinary PCA3 has recently been studied extensively for the prediction of prostate biopsy results and treatment outcomes. Current data suggests that PCA3 is particularly useful to select patients for which the biopsy should be repeated when the first biopsy turns out negative (6). It is thus considered as a complement of PSA in the detection and management of early PCa. Recent works also reveal that PCA3 is involved in the control of PCa cell survival and modulates androgen receptor (AR) signaling (7).

Circulating microRNAs in blood of PCa patients present an additional type of potential biomarkers (8). One of the promising miRNAs in advanced PCa is miR-141 which has emerged as a potential circulating diagnostic and prognostic marker across independent studies including ours (9-13). Recent work also shows that miR-141 is androgenregulated and modulates transcriptional activity of the androgen receptor (14, 15).

In this study we designed an experimental cell model with androgen-sensitive LNCaP cells to comparatively assess the extent of androgen responsiveness of three PCa-related RNA molecules (PCA3, miR-14 and PSA-mRNA). Mimicking in vivo conditions, we further examined how androgen stimulation of PCa cells affects the release of these molecules into culture medium. To confirm their release from cells *in vivo*, these molecules were also measured in the blood plasma of the patients with early PCa which is considered to be analogous to androgen-responsive cells. 

## Materials and Methods

### Culture and hormone treatment of LNCaP cells

This study includes experimental research conducted on human prostate adenocarcinoma LNCaP cells originally obtained from the American Type Culture Collection (Manassas, VA, USA). LNCaP cells were routinely maintained in the regular medium (RPMI 1640 medium supplemented with 10% fetal bovine serum (FBS). Cells were seeded at a density of 1×10^5^ cells in culture plates and grown for 48 hours in a medium supplemented with FBS treated with dextran-coated charcoal-treatment (Sigma-Aldrich, Zwijndrecht, Netherlands) according to the manufacturers’ instructions. This step aims to ablate steroid hormones in culture medium. The medium was then replaced by a fresh one containing dihydrotestosterone (DHT, C19H30O_2_) (Sigma-Aldrich, USA) at concentrations of 0, 1, 10 or 100 nM, and the cells were then allowed to grow for a further 24 hours. The cells and media were then harvested and stored for subsequent analysis.

### Patient samples

A cohort of 34 patients with pathologically confirmed early PCa and 15 healthy men were enrolled in the study (Table 1). Patients received no treatment at the time point of blood withdrawal. Our study was carried out in accordance with the Declaration of Helsinki (2008) of the World Medical Association and was approved by the Local Ethics Committee of the Istanbul Medical Faculty (No.2011/1476). Each patient gave informed consent. Plasma was immediately separated from blood cells and stored at -80˚C for subsequent analyses.

**Table 1:** Characteristics of patients recruited in this study


	N	Medianage	Median PSA value (ng/mL)	Median gleason score

**Patients**	34	65	5.23	6
**Healthy controls**	15	60	NA	NA


NA; Not applicable and PSA; Prostate specific antigen.

### RNA extraction and quantitative polymerase chain reaction (qPCR)

All RNA molecules were measured from total RNA fraction of cellular nuclear RNA, culture medium and blood plasma. To isolate total RNA, we used a monophasic phenol and guanidine thiocyanate solution (Roche, Mannheim, Germany) according to the manufacturer¡¯s protocol and 200 μl of the culture medium. Isolated RNA was dissolved in PCR-grade water. cDNA was produced using the miScript II RT Kit (Qiagen, Valencia, CA, USA) which simultaneously converts all RNA species into cDNA. Therefore, miRNAs, and PSA and PCA3 mRNA were amplified from the same sample. To quantitate miRNAs we used miScript Primer Assays (Qiagen, USA) which include a universal primer specific to the poly-A tail of the miRNA and a miRNA-specific primer. SYBR Green (Qiagen, USA) was used as the florescent molecule. The amplified PCR product had a size of approximately 80 bp. The miR-16 molecule was used as an internal control since it has been used as a reference gene by several articles (16, 17). This is because of its consistent expression in biological fluids and relatively stable expression during androgen treatment of various doses in LNCaP cells and in blood plasma in our study. For PCA3 and PSA-mRNA, GAPDH was used as the internal control and the following primer pairs were used; PCA3-F 5'-GGTGGGAAGGACCTGATGATAC-3', PCA3- R 5'-GGGCGAGGCTCATCGAT-3'; PSA-mRNA-F 5'-TGAACCAGAGGAGTTCTTGAC-3', PSA-mRNA- R 5'-CCCAGAATCACCCGAGCAG-3'; GAPDH¨C F 5'-GCTCTCTGCTCCTCCTGTTC-3' and GAPDH¨CR 5'- ACGACCAAATCCGTTGACTC-3'. Real-time PCR (RT-PCR) was performed using the LightCycler 480 Instrument (Roche, Germany). Amplification of the appropriate product for each molecule was confirmed by melting curve analysis. Expression levels of each RNA are originally presented as threshold cycle (Ct) values, defined as the fractional cycle number at which the fluorescent signal exceeds the fixed threshold in qRT-PCR. For data analysis, we used the comparative Ct method (cCt), normalized by subtracting the Ct value of endogenous reference from that of each RNA. Data for each RNA were then summarized as the mean value of cCt.

### Statistical analyses

We assessed the results of at least five independent cell culture experiments to calculate the expression of miR-141, and PCA3 and PSAmRNA in cells and culture medium. Changes relative to basal levels were expressed as "fold changes" and median values were statistically compared by the median test. Correlations between the expression of molecules were analyzed using the Pearson correlation test while the differences of their expression in plasma samples were assessed using the Mann-Whitney U test. P<0.05 was considered as the level of significance.

## Results

**Fig 1:**
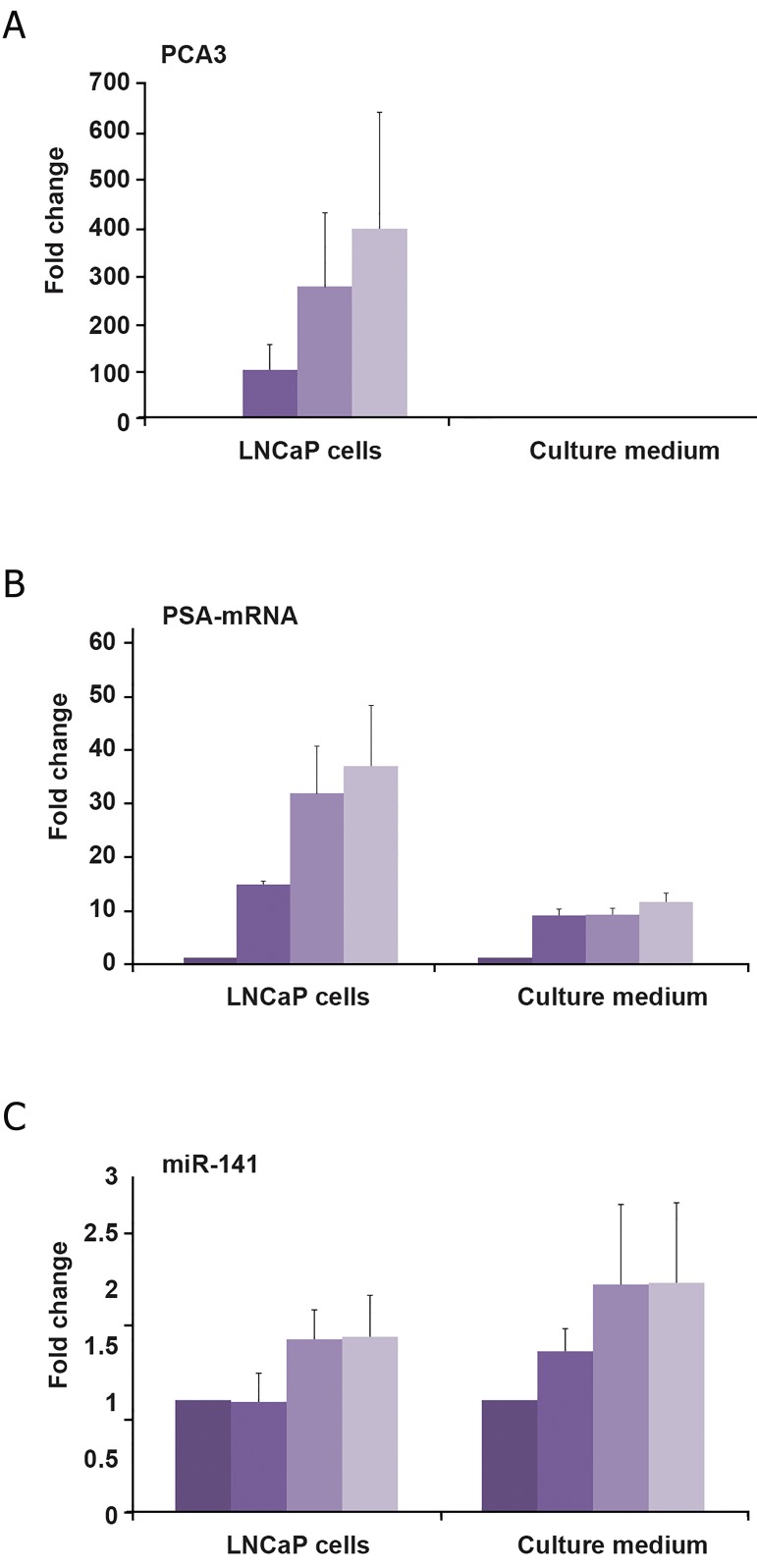
Androgen stimulation of prostate cancer antigen 3 (PCA3) (A), Prostate specific antigen (PSA) mRNA (B) and, microRNA-141 (miR-141) (C) in prostate cancer cells and their release into medium. Following hormone depletion for 48 hours LNCaP cells were treated with 0, 1, 10, and 100 nM dihydrotestosterone (DHT) for 24 hours and RNA molecules quantified in quantitative polymerase chain reaction (qPCR). GAPDH was used as the reference gene for PCA3 and PSA-mRNA while miR-16 was used as the reference gene for miR-141. Results of five independent experiments were evaluated. Basal level (0 nM DHT) of each molecule was taken 1, and changes were expressed as ‘fold changes’. Median and maximum values are shown.

Since we used the same internal control for PCA3 and PSA-mRNA, we could compare their basal expression levels to each other. Basal levels of PCA3 in LNCaP cells were approximately 12.5-fold lower than that of PSA-mRNA. As seen in figure 1, the effect of DHT on the expression of these three molecules was extremely variable. PCA3 was the most evidently stimulated RNA molecule and up-regulated up to 400-fold in mean (p˂0.001) in a dose-dependent manner (Fig 1A). Compared to PCA3, the extent of stimulation of PSA-mRNA was less distinctive where the range was 14-30-fold (p˂0.001, Fig 1B). In contrast, miR-141 was weakly induced in a dose-dependent way where the median stimulation reached only 1.32-fold (p=0.07). It is obvious that relatively small DHT dose (1 nM) is able to stimulate PCA3 and PSA-mRNA while the effect on miR-141 is observed from 10 nM.

In terms of release into culture medium, a different picture was observed. In culture medium, PCA3 was not at detectable levels even if it was highly induced by DHT (up to 400-fold). As expected, DHT treatment led to higher levels of PSA-mRNA release (9-12-fold) compared with untreated cells (Fig 1B). Similar to its induction in cells, miR-141 release was slightly induced (1.7-fold, p=0.07) by the highest DHT dose (100 nM) (Fig 1C). PSA-mRNA in medium correlated to its cellular levels (p=0.005) but this did not apply to miR-141.

**Fig 2:**
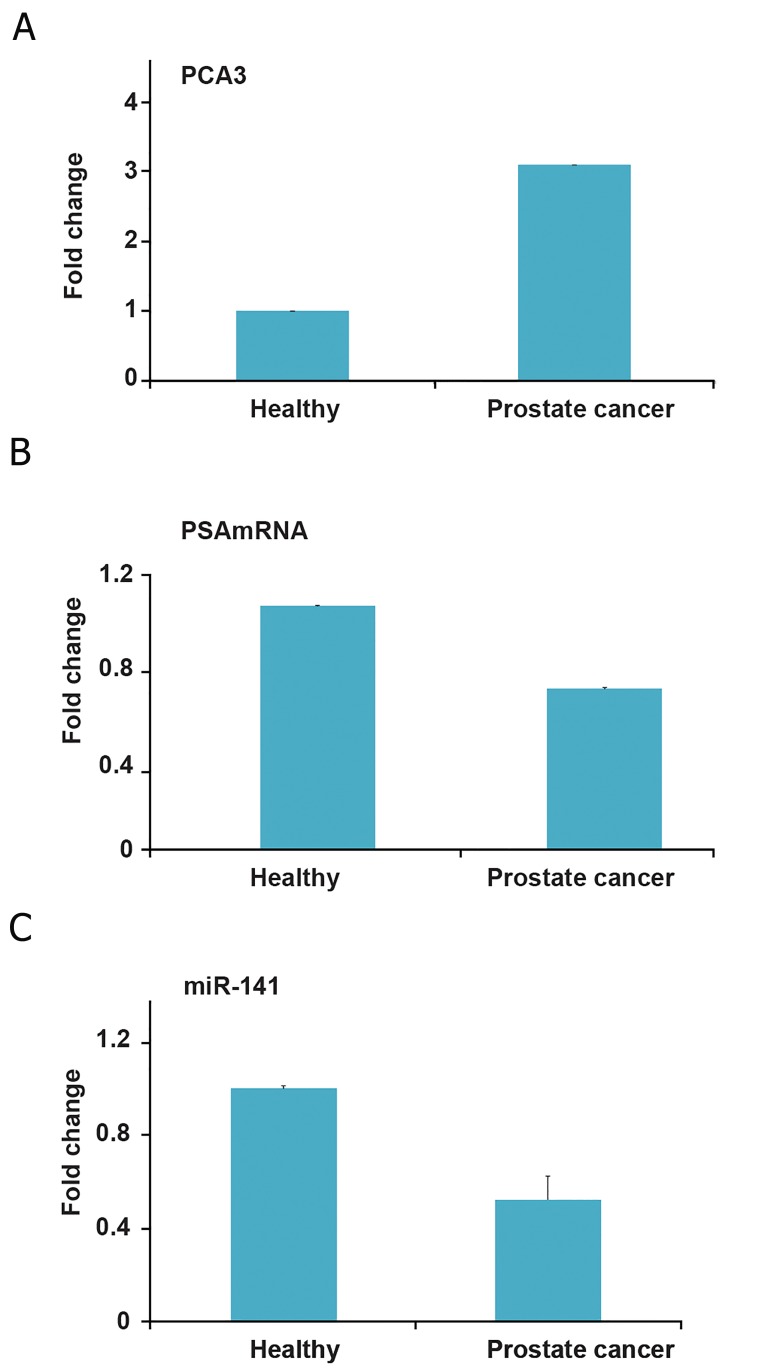
Plasma levels of prostate cancer antigen 3 (PCA3) (A) and prostate specific antigen (PSA) mRNA (B), and micro- RNA-141 (miR-141) (C) in prostate cancer (Pca) patients. Total RNA was isolated from plasma, and following cDNA synthesis, each RNA molecule was relatively quantified. Expression levels in healthy individuals were taken as 1 and changes in patients were expressed as 'fold changes'.

Next we analyzed the plasma levels of these molecules in PCa patients and healthy subjects. Similar to its undetectable levels in culture medium, PCA3 was present at low concentrations in plasma (Fig 2A), however, the patients had significantly higher levels of PCA3 than healthy controls (p=0.001). Circulating PSA-mRNA and miR-141 levels were similar between the study groups (Fig 2B, C, p=0.9).

## Discussion

In this study, we assessed the extent of androgen responsiveness of three PCa-related RNA molecules (PCA3, PSA-mRNA and miR-141) and their release from cells into culture medium. Our results show that DHT differentially affects the expression of these molecules in LNCaP cells. Of these three molecules, PCA3 was remarkably stimulated while the effect on PSA-mRNA was moderate. When compared to PCA3 and PSA-mRNA, androgen stimulation of miR-141 was relatively weak. In a recent report (14) a similar rate of androgen stimulation has been described for miR- 141. Considering its prostate-specific expression (5) and role in the modulation of AR target genes (7), PCA3 appears to be an essential component of AR signaling which may require higher levels of stimulation than PSA or miR-141. In line with this assumption, unlike miR-141, even a small dose of DHT (1 mM) was effective to strongly induce PCA3 (approx 100-fold). In addition, significantly higher plasma levels of PCA3 in PCa patients indicate its specificity for PCa. In accordance with this, a recent paper from Neves et al. (18) has described the detection of PCA3 in blood.

We were also interested in how androgen stimulation affects the release of these molecules into culture medium. PCA3 was not at detectable levels in culture medium despite very strong induction by DHT. We assume that this is due to low levels of basal expression. Stability could not be a reason of this as we could detect PCA3 in blood plasma which contains many RNases. Similar to its induction pattern in the LNCaP cells, miR-141 was released at a limited rate into the culture medium (up to 1.7-fold), but not significant. This effect appears small when compared with PSA-mRNA which is released in much higher amounts (up to 11-fold) consistent with previous reports for PSA protein upon androgen stimulation (19). Nevertheless, increased release of miR-141 from androgen-stimulated cells in patients with advanced PCa could show higher levels of this molecule in blood circulation (9-13). It has been reported that in those patients, several enzymes involved in the synthesis of androgens are highly expressed in tumor tissue (20).

Stability of RNA molecules in biological fluids or cell culture medium is a prominent issue. It has been shown that RNA in biological fluids is protected from degradation by its inclusion in protein or lipid vesicles (21). It could be speculated that the differences between PCA3 and PSA mRNA or miR-141 in their amounts in cell culture medium or patients’ blood may be related to inclusion in those vesicles such as exosomes or microvesicles (MVs). Several studies have described concentrated levels of RNA in exosomes (22).

## Conclusion

Our findings suggest a differential androgen regulation for PCa-related RNA molecules. PCA3 was the most evidently induced molecule by DHT. Elevated expression of PSA mRNA in cells is associated with their increased release into culture medium while PCA3 could not be amplified from culture medium. Further studies are required to elucidate possible mechanisms of the differential androgen responsiveness between these molecules and the clinical utility of PCA3 detection in plasma.
